# Giant Morpheaform Basal Cell Carcinoma Mimicking Scarring Alopecia: Exception Prone to Neglect

**DOI:** 10.3390/dermatopathology11020016

**Published:** 2024-06-05

**Authors:** Carlo Francesco Tomasini, Giacomo Fiandrino, Emanuele Mario Favale, Francesca Antoci, Stefania Barruscotti

**Affiliations:** 1 Dermatologic Clinic, Fondazione IRCCS Policlinico San Matteo, 27100 Pavia, Italy; emanuelemario.favale@gmail.com (E.M.F.); s.barruscotti@smatteo.pv.it (S.B.); 2 Department of Clinical, Surgical, Diagnostic and Pediatric Sciences, Institute of Dermatology, Università degli Studi di Pavia, 27100 Pavia, Italy; 3 Pathology Unit, Fondazione IRCCS Policlinico San Matteo, 27100 Pavia, Italy; g.fiandrino@smatteo.pv.it (G.F.); f.antoci@smatteo.pv.it (F.A.)

**Keywords:** basal cell carcinoma, scalp, giant, morpheaform, alopecia neoplastica, hedgehog inhibitors

## Abstract

A 74-year-old woman in good general health presented with a 5-year history of progressive hair loss over several years, interpreted as female androgenetic alopecia (AGA), and was treated with topical 5% Minoxidil without improvement. The patient’s relevant medical history revealed infiltrating, triple-negative apocrine carcinoma of the right breast four years before, treated by quadrantectomy, radiation, lymphadenectomy and chemotherapy, with no recurrence at the last follow-up. On examination, there was an asymptomatic 15 × 15 cm firm and whitish area of scarring alopecia on the central scalp. Dermoscopy revealed multiple arborizing vessels and many telangiectasia. The clinical considerations included mainly cutaneous metastasis of breast carcinoma (alopecia neoplastica), pseudopelade of Broque and morpheaform basal cell carcinoma (BCC). A histopathologic examination revealed characteristic changes of morpheaform BCC with basaloid islands and cords of atypical basaloid cells diffusely infiltrating the dermis, embedded in a sclerotic and hypervascularized stroma. Secondary alopecia neoplastica due to morpheaform BCC on the scalp is an exceedingly rare entity, possessing subtle clinical features that may mimic both scarring and non-scarring alopecia. Delayed recognition may contribute to aggressive behavior and extensive local destruction. Treatment with hedgehog inhibitors in locally advanced BCC of the scalp, both in adjuvant and neoadjuvant modalities, is promising.

## 1. Introduction

Alopecia neoplastica (AN) is a rare manifestation of cutaneous metastasis to the scalp from primary visceral malignant tumors, commonly the breast, gastrointestinal, kidney or lung [1,2]. Its clinical presentation is an asymptomatic, usually localized, skin-colored or pinkish plaque of scarring alopecia. First described by Ronchese in 1949 [3], the term “AN” was coined by Cohen et al. in 1961, who reported three additional cases in women with breast cancer (BC) presenting scalp metastases as areas of scarring alopecia [4]. Albeit rarely, AN may be due to a primary scalp neoplasm, including desmoplastic melanoma [5], basal cell carcinoma (BCC) [6], angiosarcoma [7], hemangioendothelioma [8], syringomatous carcinoma [9], microcystic adnexal carcinoma [10], ectopic extramammary Paget’s disease [11], and B-cell lymphoma [12]. The underlying mechanisms of AN still remain to be clearly defined. It has been postulated that neoplastic cells destroy hair follicles by inducing fibroplasia through the production and release of cytokines and interleukins that recruit inflammatory cells and replace the normal cell population [12]. However, the infiltration of neoplastic cells may, at times, not be apparent or only minimally—a condition known as “scalp alopecia due to a clinically unapparent or minimally apparent neoplasm” (SACUMAN) [13]. In this case, there may be a clinical suspicion of an inflammatory or hormonal alopecia, but this suspicion is invalidated when the clinical manifestations become striking.

Herein, we report on a case of an elderly woman with BC who presented with a long-standing wide area of scalp alopecia that was misinterpreted as androgenetic alopecia and actually turned out to be morpheaform BCC.

## 2. Case Report

**Figure 1:**
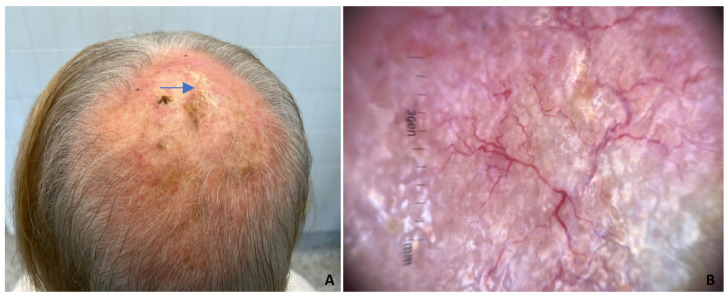
(**A**) A large, translucent, shiny and waxy alopecic plaque with ill-defined margins and marked neovascularization on the central scalp. The crusted area is due to a previous punch biopsy (arrow). (**B**) Dermoscopy evidences multiple arborizing vessels, porcelain white areas and complete loss of follicular openings.

**Figure 2:**
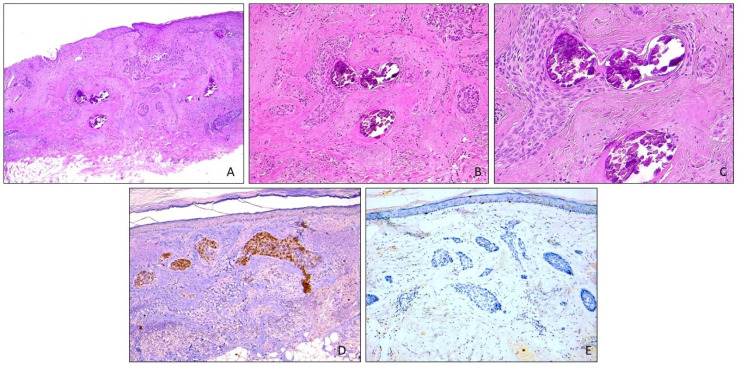
(**A**,**B**) Full dermal infiltration of nests and strands of basaloid cells with focal calcification. The stroma is dense and sclerotic. (**C**) Closer view showing calcium deposits within neoplastic aggregates. (**D**) Strong and diffuse immunostaining for Ber-EP4. (**E**) EMA expression is negative.

A 74-year-old woman who was in good general health was referred to our department for progressive hair loss for more than 5 years. The condition had been diagnosed elsewhere as female androgenetic alopecia and treated unsuccessfully with topical 5% Minoxidil, whilst the bald area had slowly continued to enlarge. The dermatological examination revealed an asymptomatic, whitish, 15 × 15 cm central scalp area with complete hair loss (Figure 1A). The plaque was hard with ill-defined borders and marked neovascularization and fine telangiectasia. There were no erosions, pustules or crusts. Dermoscopy revealed multiple arborizing vessels, structureless hypopigmentation and a complete loss of follicular openings (Figure 1B). The patient’s clinical history revealed infiltrating, triple-negative apocrine carcinoma of the right breast that had been treated by quadrantectomy, axillary lymphadenectomy, radiotherapy and chemotherapy four years before. At her last follow-up, she was negative for recurrence. There was no history of scalp radiotherapy or pre-existing chronic cutaneous lesions. A histopathologic examination of a punch biopsy from the alopecic plaque revealed basaloid islands and cords of atypical basaloid cells that diffusely infiltrated the dermis and extended into the subcutis, embedded in a sclerotic and hypervascularized stroma (Figure 2A). Peripheral palisading and retraction of clear spaces were inconspicuous. Some basaloid aggregates had central calcium deposits (Figure 2B,C).

**Figure 3:**
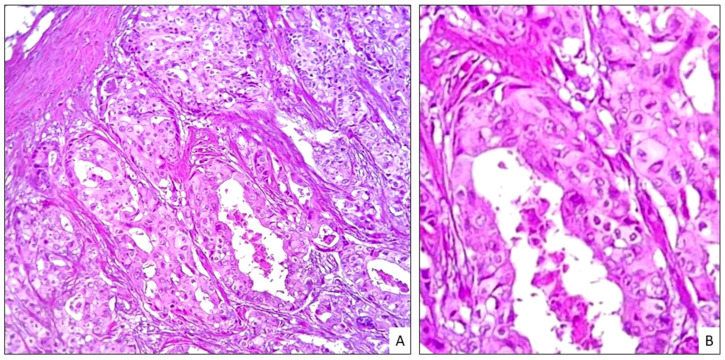
(**A**) Apocrine breast carcinoma showing large atypical epithelial cells with abundant eosinophilic and granular cytoplasm, enlarged nuclei and prominent nucleoli. (**B**) High magnification shows pleomorphic discohesive neoplastic cells and cystic necrosis.

As the main concern was to definitely rule out secondary AN, archival material from her previous breast carcinoma was retrieved for a combined cytomorphology and immunohistochemistry comparison. The primary BC had characteristic apocrine morphology of neoplastic cells with abundant eosinophilic and granular cytoplasm, well-defined cell borders, enlarged round nuclei and prominent nucleoli with foci of comedo necrosis (Figure 3A,B), completely different from the basaloid character of the scalp neoplasm. Although both neoplasms expressed CK5/6 and Ber-EP4 (Figure 2D), the scalp neoplasm was negative for EMA (Figure 2E), CK7, racemase, mammaglobin and GCDF15. Three additional skin biopsies were carried out to evaluate the lateral extension of the tumor and revealed histopathological changes superimposable to those observed in the first biopsy and were consistent with morpheaform BCC. Mammography, ultrasound examination of the breasts and a total body computed tomography (CT) scan were negative for BC recurrence. There was no evidence of galea infiltration on the skull magnetic resonance imaging (MRI). The patient was referred to our multidisciplinary team, who decided to perform surgical removal of the entire plaque after neoadjuvant treatment with the hedgehog (Hh) inhibitor Sonidegib, aimed at achieving a preoperative size reduction of the neoplasm.

To analyze the tumor mutation profile, the AmoyDx^®^ HANDLE Classic NGS Panel (Amoy Diagnostics Co., Xiamen, China) was also used, and DNA and RNA were isolated from formalin-fixed paraffin-embedded (FFPE) tumor tissue. No mutations of pathogenetic significance were detected.

## 3. Discussion

BCC is the most frequent malignant neoplasm in white-skinned individuals. Despite its high prevalence, etiopathogenesis is multifactorial, and UV radiation is the main causative factor [14]. Whilst the head and neck are the most common locations for BCC, accounting for 85% of cases [14], scalp BCC prevalence varies from 1.1% to 2.7% in a large series, respectively, from Brazil [15] and Australia [16]. In a smaller Italian series, the prevalence of scalp BCC is 13% [17].

Scalp BCC mainly occurs in the elderly without significant gender differences [16]. Conversely, Tosti [18] and Katz [19] reported a higher prevalence in females. Risk factors for scalp BCC include UV radiation, radiotherapy and immunosuppression, which are the same for BCC in general. However, the bimodal distribution of scalp BCC in females, with a large peak in the 40- to 49-year-old age group and a slightly smaller peak in the 70- to 79-year-old age group, would suggest that chronic UV exposure plays a lesser carcinogenetic role in scalp BCC, especially in younger patients [19]. Moreover, scalp BCCs are frequently observed on pre-existing chronic lesions, such as radiodermatitis or Jadassohn’s sebaceous nevus [20]. The most common clinicopathologic presentation is the nodular type, whilst aggressive subtypes, such as infiltrative/morpheaform and the micronodular types, account for about one-third of cases and are responsible for the larger size and highest recurrence rate [19,20].

Scalp alopecia due to a clinically unapparent or minimally apparent neoplasm (SACUMEN) is a rare condition [13]. Whilst the most common neoplasm is metastatic BC, only a few cases of BCC have been reported in the literature [6,13,15,21,22]. In our case, the gross clinical similarity to androgenetic alopecia, without pearly borders or ulceration, together with its subtle course, may explain the long tumor neglect and its large size reached at the time of diagnosis.

On examination, the scarring character of alopecia of our patient could lead to the suspicion of several causes of end-stage scarring alopecia, such as chronic discoid lupus erythematosus, lichen planopilaris, erosive pustular dermatosis of the scalp, and frontal fibrosing alopecia. The presence of arborizing vessels, structureless hypopigmentation and complete loss of follicular openings on dermoscopy, with an absence of classical dermoscopic criteria of hair disorders, was strongly suggestive of malignancy.

Dermoscopy is an especially important diagnostic tool for alopecia and other scalp disorders and may be of help to the clinician in identifying the most appropriate biopsy site [18]. It has recently been emphasized that the absence of classical criteria of other scalp diseases, along with major neovascularization with on-focus arborizing vessels, are clues of secondary AN, especially, as in our case, when assessing a patient with a history of BC [23]. However, the presence of arborizing vessels, considered almost pathognomonic of BCC, can be observed in conditions other than AN and BCC, including benign cystic, non-cystic, premalignant and malignant tumors and even in non-tumors [24]. Therefore, differentials other than BCC and secondary AN should be considered.

The multiple skin biopsies from our patient’s plaque revealed characteristic histopathologic features of morpheaform BCC. Although histopathologic differentiation between BCC and skin metastasis of BC is usually straightforward, in cases where the latter does not express hormone receptors or HER2 in the primary lesion or the metastasis, if there is marked basaloid differentiation, or the tissue has been poorly preserved, a clear cut differentiation may be challenging [25]. Immunohistochemistry may be of limited value, as often most breast-related markers can be expressed only faintly and focally by BCC, with the exceptions of mammaglobin, gross cystic disease fluid protein 15 (GCDFP-15) or α-Methylacyl-CoA racemase (AMACR; P504S)—the latter being useful when faced with apocrine BC [25]. In our case, the completely different histomorphology, along with negative EMA and positive BER-EP4 staining in the scalp neoplasm, supported a diagnosis of BCC whilst excluding other entities, such as moderately differentiated SCC or basosquamous carcinoma.

According to the American Joint Committee on Cancer, the scalp BCC of our patient falls into the category of giant BCC (GBCC), which is defined as a tumor larger than 5 cm in diameter [26]. However, some authors consider GBCC as tumors only measuring 10 cm or more [27].

GBCC is a rare oncological entity, mostly occurring in elderly males, with a peak incidence in the seventh decade of life [28]. It usually presents as a long-neglect tumor, with an average disease duration of 14.5 years. The prevalent location on the trunk, especially the back, may explain the tumor not being detected for a long time. Other risk factors for neglected tumors include the morpheaform histologic subtype, socioeconomic status and physical or psychiatric disabilities that may impair access to healthcare [29].

GBCC is an aggressive malignant neoplasm with a high rate of local recurrence or metastasis. Treatment guidelines for GBCC are not established. Wider surgical excision with histologically confirmed tumor-free margins is recommended, whilst monotherapy with chemotherapy and radiotherapy are of limited efficacy [28,30]. Whilst during the pre-hedgehog inhibitor therapy era the overall cure rate of GBCC was 61.7% at an average follow-up of 2 years [28], currently, treatment with Hh inhibitors—both in adjuvant and neoadjuvant modalities—appears to offer promising results [31,32,33].

## 4. Conclusions

Morpheaform GBCC of the scalp is an extremely rare, diagnostically challenging entity. Its clinical features may mimic both scarring and non-scarring alopecia, leading to delayed recognition and contributing to more aggressive behavior and extensive local destruction.

## Data Availability

Data are available upon request from the corresponding author.
